# Wolff-Parkinson-White syndrome: a masquerading clinical condition in an 8-year-old Nigerian girl

**DOI:** 10.1186/s12872-025-05246-x

**Published:** 2025-10-29

**Authors:** Olukemi T. Bamigboye-Taiwo, A. A. Afolabi, Oluwatosin Olorunmoteni, Samson Afolabi, Babajide Samson Adeyefa, OA Bayode, OO Bobo, EO Folami, Olayinka Otetubi, FA Olagunju, SBA Oseni, Oluwadare Ogunlade, JAO Okeniyi

**Affiliations:** 1 Paediatric Cardiology Unit, Department of Paediatrics and Child Health, Obafemi Awolowo University, Ile-Ife, Nigeria; 2 Paediatric Cardiology Unit, Department of Paediatrics, Obafemi Awolowo University Teaching Hospitals Complex, Ile-Ife, Nigeria; 3 Paediatric Cardiology Unit, Department of Paediatrics, UNIOSUN Teaching Hospital, Osogbo, Nigeria; 4 Paediatric Neurology Unit, Department of Paediatrics and Child Health, Obafemi Awolowo University and Obafemi Awolowo University Teaching Hospitals Complex, Ile-Ife, Nigeria; 5 Department of Anaesthesia, UNIOSUN Teaching Hospital, Osogbo, Nigeria; 6 Paediatric Infectious Disease Unit, Department of Paediatrics, UNIOSUN Teaching Hospital, Osogbo, Nigeria; 7 Cardiology Unit, Department of Medicine, Obafemi Awolowo University Teaching Hospitals Complex, Ile-Ife, Nigeria

**Keywords:** Case report, WPW syndrome, Congenital, Electrocardiogram, SVT, Paediatrics

## Abstract

**Background:**

Wolff-Parkinson-White (WPW) syndrome is a congenital abnormality of the cardiac conduction system characterized by the presence of an accessory pathway, which can predispose affected individuals to supraventricular tachycardia (SVT), atrial fibrillation, ventricular fibrillation, and sudden cardiac death. Despite its clinical significance, WPW syndrome is often underdiagnosed, particularly in resource-limited settings where cardiac arrhythmias may be misattributed to other conditions.

**Case presentation:**

We report an eight-year-old Nigerian girl with WPW syndrome who was repeatedly misdiagnosed and managed for malaria over four years before an accurate diagnosis was established. She presented with recurrent episodes of chest discomfort, generalized weakness, nausea, and near-syncope. Each episode was treated as malaria, and symptoms resolved following treatment. During the most recent episode of the symptoms, she received care in a tertiary centre where SVT was identified following an electrocardiogram (ECG), heart rate was persistently about 250 beats per minute. Initial pharmacologic intervention with intravenous amiodarone was ineffective, necessitating external cardioversion to restore normal sinus rhythm. A post-recovery ECG confirmed a Wolf Parkinson White pattern.

**Conclusion:**

WPW syndrome remains a diagnostic challenge in paediatric populations, where it can masquerade varying diseases, resulting in misdiagnosis. This case underscores the importance of a high index of suspicion for cardiac arrhythmias in children presenting with unexplained recurrent symptoms. Early recognition and appropriate intervention are crucial in preventing life-threatening complications associated with WPW syndrome. Increased awareness among healthcare providers can lead to improved diagnostic accuracy and better patient outcomes.

## Introduction

Wolff Parkinson White (WPW) syndrome is the most common form of ventricular pre-excitation affecting 0.1–0.3% of the general population [1]. The first description of WPW syndrome was made in 1930 by Louis Wolff, Sir John Parkinson, and Paul Dudley White [2]. WPW syndrome is marked by the presence of an accessory pathway (the bundle of Kent) that travels between the atria and the ventricles creating an alternate path for the activation of the ventricles [3]. The alternate path circumvents the atrioventricular node (AVN) thereby allowing for premature activation of the ventricles i.e., pre-excitation [3]. WPW syndrome can affect all ages but is more often diagnosed in children, adolescents, and young adults [4].

###  Case presentation

The patient was four years old when her symptoms started. Even though she was verbal then, she still was unable to fully articulate what she felt. However, she was observed to have chest discomfort, followed by generalized weakness, nausea and vomiting. These were accompanied by near syncope. Duration of symptoms varied, lasting between one to four days during which she experienced extreme weakness and was unable to function or even feed. She also had an intercurrent fever. Following the onset of symptoms, she would present in a Clinic facility where the diagnosis of malaria was made each time. She was admitted rehydrated and treated accordingly. Resolution of symptoms followed treatment. The frequency of symptoms varied from once in three months to thrice a month. As she grew older, she was able to recognise the onset of symptoms and described it as a sudden thump in her chest. She referred to it as “tolotolo” which in the local language meant ‘turkey” Once she felt a thump in her chest, she would inform her parents that her symptoms were about to start. Parents noticed she would quieten herself and within 30 minutes the full symptoms occurred. After four years of recurrence of these episodes, her parents requested a referral to a tertiary facility. General and systemic examination did not reveal any abnormality, she had a normal weight and a normal height and initial investigations were normal. Echocardiogram shows normal cardiac structure and function. The symptoms described seemed to fit into a form of seizure disorder. On further investigations, an electroencephalogram as well as a brain magnetic resonance imaging scan came back normal. During the most recent episode of the symptoms, she received care in a tertiary centre where her heart rate persistently ranged between 224–250 beats per minute. An electrocardiogram (ECG) revealed supraventricular tachycardia. She had a carotid sinus massage which brought a very temporary relief, only for the heart rate to return to 250 bpm after a few minutes. The application of a cold compress to the face did not help. She was managed with intravenous amiodarone. Despite this, the tachyarrhythmia persisted. She was subsequently scheduled for cardioversion using an automated external defibrillator (AED) on the fourth day of admission. Multiparameter monitoring was attached, with continuous recording of pulse rate, oxygen saturation, temperature, and blood pressure. She was connected to a defibrillator (the team ensured a safe distance to prevent accidental shocks). Intravenous midazolam 1 mg was administered, and the AED pads were attached. The device automatically analyzed the patient’s heart rhythm, which was assessed to be shockable. A shock was then delivered at 100 joules. Prior to the shock, the patient’s pulse rate was recorded at 234 beats per minute. Following the cardioversion, the rhythm successfully reverted to 84 beats per minute, with immediate normalization of the heart rate. ECG following recovery showed characteristic features of Wolff Parkinson White syndrome. She has remained stable since then and has not had another episode up until the time of this report.

##  Discussion

**Fig. 1:**
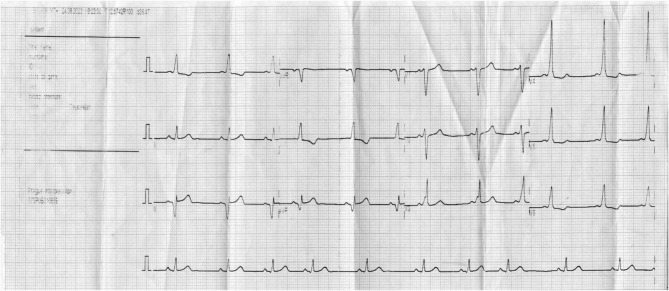
Initial electrocardiogram at first visit

Wolff Parkinson White syndrome is a rare condition and is infrequently diagnosed in children in our setting. The classical features of WPW syndrome on the electrocardiogram (ECG) are a shortened PR interval of less than 120 ms, slurred QRS upstroke (the delta wave), and a widened QRS complex of greater than 120 ms [5]. The Wolff Parkinson White syndrome is the combination of ECG changes with episodes of tachycardia [1, 5]. The ECG findings alone without documented tachyarrhythmia or related symptoms are referred to as the Wolff Parkinson White pattern [1, 6] (Figure 1).

Based on the ECG findings of the index patient which showed the classic WPW pattern, and the episode of tachyarrhythmia -supraventricular tachycardia, a diagnosis of Wolff Parkinson White syndrome was made.

**Fig. 2:**
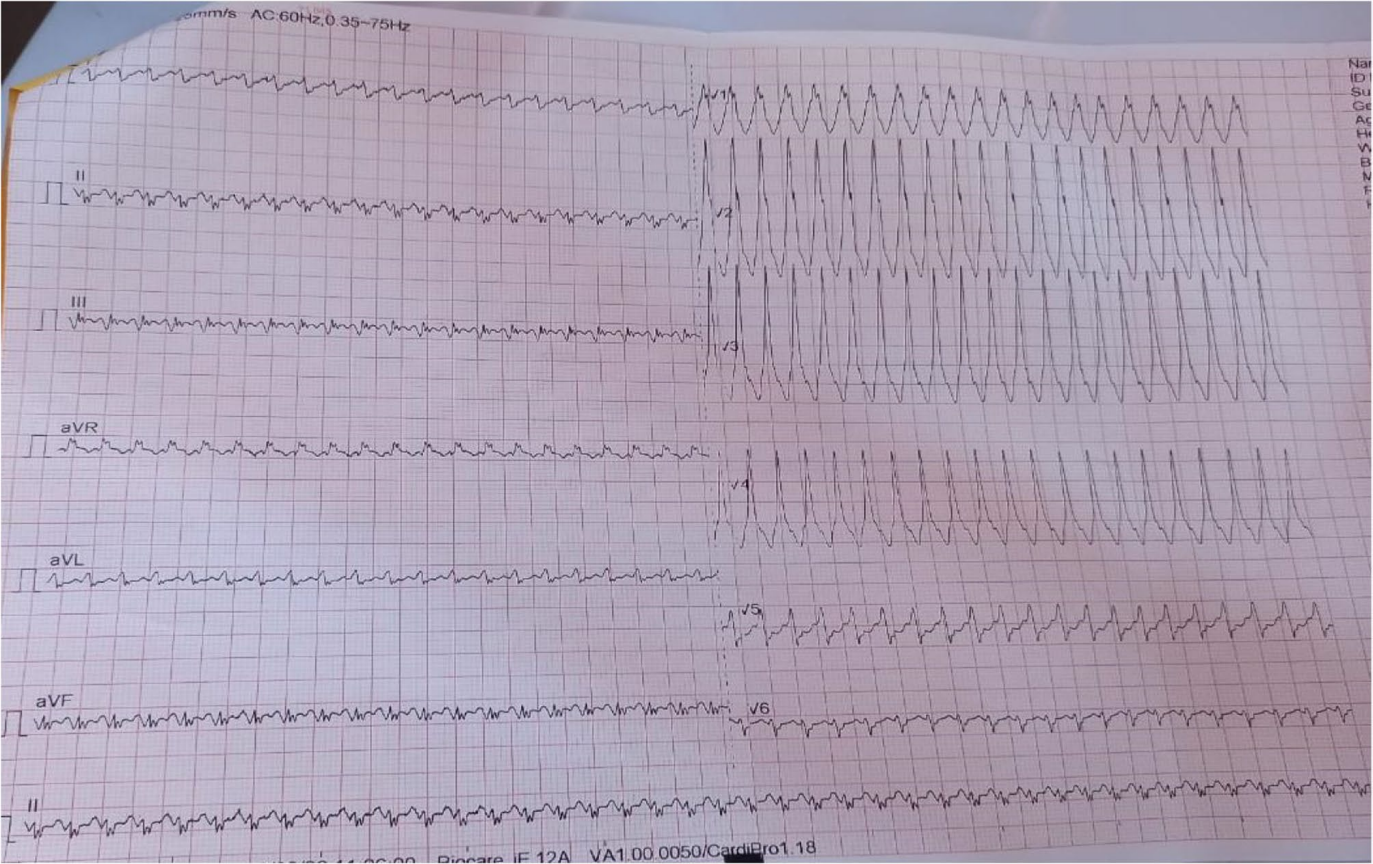
ECG during an acute episode showing supraventricular tachycardia

Patients with WPW syndrome may present with a myriad of symptoms including unexplained anxiety, dyspnoea, chest pain, palpitations, fatigue, light-headedness or dizziness, syncope and near syncope [7]. In a longitudinal study involving 446 patients all aged 20 years and below, Cain et al. [8] reported the findings at presentation included supraventricular tachycardia in 38%, palpitations in 22%, chest pain in 5%, syncope in 4%, atrial fibrillation (0.4%), sudden death (0.2%), and incidental findings (26%). It is widely reported in the literature that the majority of patients remain symptom-free all through their lifetime while approximately half of the patients with WPW syndrome experience tachyarrhythmias [6, 7] (Figure 2).

**Fig. 3:**
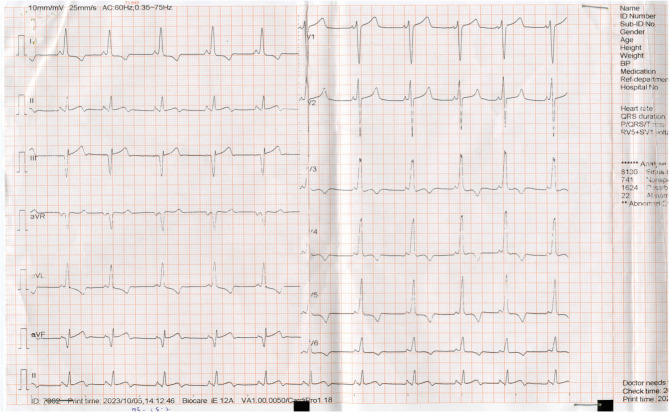
ECG following resolution of symptoms showing Wolff Parkinson White pattern

Wolff Parkinson White syndrome can masquerade, and diagnosis may remain elusive. The index patient had been managed repeatedly for malaria for four years before the diagnosis of WPW syndrome was finally made. Madal [4] reported on an adult patient who was managed for anxiety disorder for eight years before a diagnosis of WPW syndrome was eventually made. Ventricular preexcitation may not be immediately obvious and thus there is the likelihood of delayed diagnosis in persons with concealed preexcitation at the time of the ECG [9]. This is more so in the absence of symptoms that indicate the presence of an accessory pathway or Wolf Parkinson White syndrome. It is important to note that the diagnosis of WPW does not require the presence of ventricular preexcitation on all twelve ECG leads [9] (Figure 3).

The relationship between WPW syndrome and sudden cardiac death (SCD) has been known for almost a century now [10].

Studies from the late 20th century indicate that the mechanism of SCD in patients with WPW syndrome involved the rapid conduction of atrial fibrillation through the accessory pathway resulting in ventricular fibrillation [11, 12].

The incidence of in SCD with WPW syndrome is approximately 0.25% per year, or in the range of 3% to 4% in a lifetime [13]. Despite being quite rare and unusual, sudden death may be the first sign of preexcitation syndrome, especially among the paediatric age group [14]. Munger et al. [15] studied 113 children and adults, and reported 2 sudden deaths with an incidence of 1.5 per 1,000 patient-years, Cain et al., reported an overall incidence of sudden death of 1.1 per 1,000 patient-years in patients with anatomically normal hearts [8]. Concern about the risk of sudden death has a significant impact on the clinical management of patients with WPW syndrome [8].

The treatment of WPW syndrome requires individualized care. There is evidence that the risk of SCD in symptomatic preexcited patients differs from the risk of SCD in asymptomatic preexcited patients [9]. Recent studies have recommended that patients who have only the WPW pattern on ECG but are asymptomatic do not require any form of treatment [14]. These patients should be counselled on their potential risk for arrhythmia and sudden cardiac death. Even though the overall risk of sudden cardiac death is minimal, it is not zero thus risk assessment and routine monitoring for this category of patients is necessary [14].

The probability of SCD in patients with ventricular preexcitation is well established. However, the exact risk in each patient depends on the electrophysiologic properties of the accessory pathway [9]. For asymptomatic patients with ventricular preexcitation, the goal of management is to adequately counsel the patient on their options and lifetime risks, especially for SCD [9].

Obeyesekere et al. [13] in a recent meta-analysis on the risk of SCD in patients with asymptomatic preexcitation reported the risk of SCD in children as 1.93 events per 1000 person-years and in adults the risk was 0.86 events per 1000 years. In all, the risk of SCD was low in all patients with asymptomatic ventricular preexcitation. The study noted that children are at increased risk of SCD and concluded that paediatric patients with ventricular preexcitation should be closely followed up and monitored for arrhythmias.

In a review of paediatric patients who underwent electrophysiologic studies, Yıldırım et al. noted that the prognosis of WPW in children is not as benign as previously thought and concluded that all patients with a WPW pattern should have electrophysiological assessment and risk-stratification [14]. Ablation of the accessory pathway in patients with risk factors can prevent sudden cardiac death in this population [14].

Symptomatic patients with arrhythmias would need electrophysiology studies to identify and map out the accessory pathway [16]. The accessory pathway can be eliminated by radiofrequency ablation. Rarely, WPW syndrome may be treated with antiarrhythmic medications, although most patients opt for the catheter ablation procedure because it is curative and eliminates the need for lifelong medication therapy [16].

The challenge of managing this condition in a developing nation like Nigeria is enormous. Facilities for electrophysiological studies are scarce. Often times, patients with WPW syndrome have to seek expert care in other countries where facilities for risk assessment and radiofrequency ablation are available.

## Conclusion

Wolf Parkinson White syndrome does occur in Nigerian children. Physicians should have a high index of suspicion. Patients may need to have repeated ECG studies, especially those with concealed pre-excitation before the classic features are identified on the electrocardiogram.

## Data Availability

No datasets were generated or analysed during the current study.

## Abbreviations

• WPW: Wolff–Parkinson–White
• SVT: Supraventricular Tachycardia
• ECG: Electrocardiogram
• AVN: Atrioventricular Node
• SCD: Sudden Cardiac Death

## References

[1] Kashaou A, Wackel P, Kowlgi GN. Asymptomatic Ventricular Preexcitation (Wolff-Parkinson-White Pattern): When to Be Concerned. Expert Analysis. American College of Cardiology. 2022. Available at: https://www.acc.org/Latest-in-Cardiology/Articles/2022/02/17/13/25/Asymptomatic-Ventricular-Preexcitation.

[2] Wolff L, Parkinson J, White PD. Bundle-Branch block with short P-R interval in healthy young people prone to paroxysmal tachycardia. Ann Noninvasive Electrocardiol. 2006;11(4):340–53. 10.1111/j.1542-474x.2006.00127.x. 17040283 10.1111/j.1542-474X.2006.00127.x PMC6932258

[3] Keating Morris FP, Brady WJ. Electrocardiographic features of Wolff-Parkinson-White syndrome. Emerg Med J. 2003;20:491–3. 12954704 10.1136/emj.20.5.491 PMC1726185

[4] Mandal A. Wolff-Parkinson-White syndrome: the supreme master of camouflage. Int J Res Med Sci. 2023;11(10):3883–388. 10.18203/2320-6012.ijrms20233053.

[5] Kulig J, Koplan BA. Wolff-Parkinson-White Syndrome and Accessory Pathways. Circulation. 2010;122:e480–e483. Available at: 10.1161/CIRCULATIONAHA.109.929372Circulation. 10.1161/CIRCULATIONAHA.109.929372 20937983

[6] Weitz D, Kulick DL, Shiel WC Jr. Wolff-Parkinson-White Syndrome (WPW).Medicine net. Updated: 5/11/2023. Available: https://www.medicinenet.com/wolff-parkinson-white_syndrome/article.htm.

[7] Madabushi R, Agarwal A, Tewari S, Gautam SKS, Khuba S. Depression masquerading as chest pain in a patient with Wolff Parkinson white syndrome. Korean J Pain. 2016;29(4):262–5. 27738505 10.3344/kjp.2016.29.4.262 PMC5061643

[8] Cain N, Irving C, Webber S, Beerman L, Arora G. Natural history of Wolff-Parkinson-White syndrome diagnosed in childhood. Am J Cardiol. 2013;112(7):961–5. 10.1016/j.amjcard.2013.05.035. 23827401 10.1016/j.amjcard.2013.05.035

[9] Mohan S, Balaji S. Management of asymptomatic ventricular preexcitation. Indian Pacing Electrophys J. 2019;19(6):232–9. 10.1016/j.ipej.2019.10.001. 10.1016/j.ipej.2019.10.001 PMC6904806 31669128

[10] Fitzsimmons PJ, McWhirter PD, Peterson DW, Kruyer WB. The natural history of Wolff-Parkinson-White syndrome in 228 military aviators: a long-term follow-up of 22 years. Am Heart J. 2001;143(3):530–6. https://api.semanticscholar.org/CorpusID:9073730. 10.1067/mhj.2001.117779 11526369

[11] Dreifus LS, Haiat R, Watanabe Y, Arriaga J, Reitman N. Ventricular fibrillation: a possible mechanism of sudden death in patients and Wolff-Parkinson-White syndrome. Circulation. 1971;43:520–7. 5573385 10.1161/01.cir.43.4.520

[12] Klein GJ, Bashore TM, Sellers TD, Pritchett EL, Smith WM, Gallagher JJ. Ventricular fibrillation in the Wolff-Parkinson-White syndrome. N Engl J Med. 1979;301:1080–5. 492252 10.1056/NEJM197911153012003

[13] Obeyesekere M, Gula LJ, Skanes AC, Leong-Sit P, George J, Klein MD. Risk of sudden death in Wolff- Parkinson-White Syndrome; how high is the risk? Circulation. 2012;125:659–60. 10.1161/CIRCULATIONAHA.111.085159. 22215858 10.1161/CIRCULATIONAHA.111.085159

[14] Yildirim I, Ozer S, Karagoz T, Sahin M, Ozkutlu S, Alehan D, Celiker A. Clinical and electrophysiological evaluation of pediatric Wolff-Parkinson-White patients. Anatol J Cardiol. 2015;15(6):485–90. 10.5152/akd.2014.5462. 26006136 10.5152/akd.2014.5462 PMC5779142

[15] Munger TM, Packer DL, Hammill SC, Feldman BJ, Bailey KR, Ballard, et al. A population study of the natural history of Wolff-Parkinson-White syndrome in olmsted County, Minnesota. 1953e1989. Circulation. 1993;87:866e873. 8443907 10.1161/01.cir.87.3.866

[16] Etheridge SP, Escudero CA, Blaufox AD, Law IH, Dechert-Crooks BE, Stephenson EA, et al. Life-Threatening event risk in children with Wolff-Parkinson-White syndrome: A multicenter international study. JACC Clin Electrophysiol. 2018;4:433–44. 30067481 10.1016/j.jacep.2017.10.009

